# Exploring the pharmacological mechanism of *Tripterygium wilfordii* hook for treatment of Behcet’s disease using network pharmacology and molecular docking

**DOI:** 10.1097/MD.0000000000034512

**Published:** 2023-10-20

**Authors:** Lihua Ye, Changrong Li, Xiaoxia Zhao, WeiHong Ou, Li Wang, Mengjie Wan

**Affiliations:** a Department of Dermatology, Affiliated Haikou Hospital of Xiangya Medical College, Central South University, Hainan, China; b Medical Cosmetology Clinic, Hainan Yilimei Medical Cosmetology Co., Hainan, China.

**Keywords:** antioxidant, Behcet’s disease, molecular docking, network pharmacology, traditional Chinese medicine, *Tripterygium wilfordii* hook

## Abstract

*Tripterygium wilfordii* hook (TWH) has been used to treat Behcet’s disease (BD) but its underlying mechanism remains unclear. This study aims to explore the mechanism of TWH on BD using network pharmacology and molecular docking. The bioactive constituents of TWH and their corresponding target genes were extracted from the Traditional Chinese Medicine systems pharmacology database and analysis platform. BD target genes were obtained by searching the DisGeNet and GeneCards databases. Gene ontology annotation and Kyoto encyclopedia of genes and genomes pathway enrichment analysis were conducted to elucidate the function of overlapping genes between TWH and BD target genes. A protein-protein interaction network was constructed using Cytoscape and STRING platforms, and the core target genes were identified from the overlapping genes. Finally, molecular docking was used to assess the binding affinity between the core targets and TWH bioactive constituents. We identified 25 intersection genes related to both TWH and BD and 27 bioactive ingredients of TWH. Through analysis of protein-protein interaction network, 6 core targets (TNF, IFNG, prostaglandin-endoperoxide synthase 2, NOS2, VCAM-1, and interleukin-2) were screened out. Enrichment analysis demonstrated that the antioxidant properties of TWH constituents might play a significant role in their therapeutic effects. Molecular docking revealed high binding affinity between the bioactive constituents of TWH, such as kaempferol, triptolide, 5, 8-Dihydroxy-7-(4-hydroxy-5-methyl-coumarin-3)-coumarin, and their corresponding target genes, suggesting the potential of TWH to treat BD. Our investigation clarified the active components, therapeutic targets of BD in the treatment of TWH and provided a theoretical foundation for further researches.

## 1. Introduction

Behcet’s disease (BD) is a chronic systemic disorder characterized by recurrent oral and genital ulceration, eye disease, vasculitis and parenchymal neurological disease.^[1]^ The epidemiology of BD is uniquely distributed in countries along the ancient Silk Road including Turkey, Iran, China, and Japan. Among the endemic countries, Turkey has reported the highest prevalence of BD with 80 to 370 cases per 100,000 population.^[2]^ Despite genetic susceptibility (HLA-B51)^[3]^ and several inflammatory pathways^[4]^ have been reported to participate in BD development, the pathogenesis of BD has not been completely elucidated. Currently, immunosuppressive agents (cyclosporin, cyclophosphamide, and azathioprine), anti-TNF antibodies (etanercept and infliximab), phosphodiesterase 4 inhibitor (apremilast), colchicine, and interferon-α are among the treatments for BD.^[5]^ However, long-term use of these treatments for BD may cause severe adverse effects such as infections, renal dysfunction, hepatotoxicity, bone marrow suppression and depression.^[6,7]^ Therefore, complementary and alternative medicine options, particularly herbal medicine, have gained attention in recent years as a potential treatment for BD and several formulas have been found to be effective and safe for BD.^[8,9]^

*Tripterygium wilfordii* hook (TWH), also known as Lei Gong Teng or thunder god vine, is one of the most representative traditional Chinese herbs with anti-inflammatory and immunosuppressive effects. It is commonly used to treat various autoimmune diseases, such as rheumatic arthritis,^[10]^ renal disease,^[11]^ inflammatory bowel diseases^[12]^ and BD.^[13,14]^ *Tripterygium glycoside*, an extract of TWH, is included in the Chinese National Essential Drug List^[15]^ and is a typical drug derived from traditional Chinese herbs with indications for BD. A study found that after 3 months of treatment with Tripterygium glycosides, the total effective rate in the BD group was 86.6% and serum levels of IL-1β, TNF-α, interferon-γ (IFN-γ), erythrocyte sedimentation, and C-reactive protein in patients with BD were significantly reduced.^[14]^ In addition, et al found that nitric oxide, soluble intercellular adhesion molecule-1, and soluble vascular cell adhesion molecule-1 (sVCAM-1) levels in patients with BD were higher than those in the control group and the expression of NO, soluble intercellular adhesion molecule-1, and sVCAM-1 decreased significantly after 2 months of treatment with *Tripterygium wilfordii*.^[16]^ Despite several studies have explored the possible role of TWH in treating BD, the mechanisms underlying its therapeutic effects on BD have not yet been fully elucidated.

**Figure 1.**
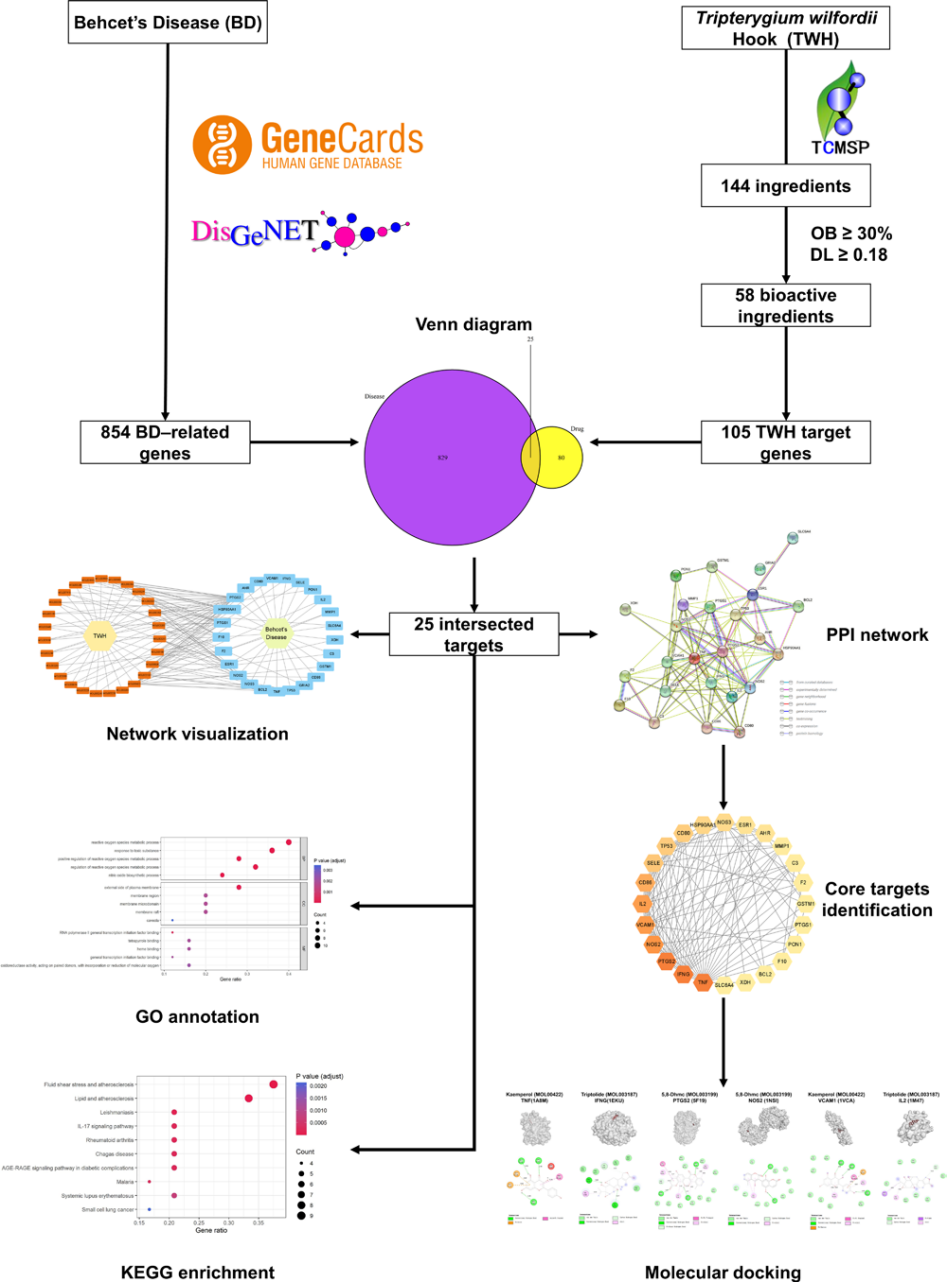
Workflow of the study design.

In contrast to modern drugs that focus on specific targets, traditional Chinese medicine, such as TWH, typically works in a multi-compound, multi-target and multi-pathway manner.^[17,18]^ The network pharmacology approach is capable of detecting complexities among drugs, genes and diseases from a network perspective, hence it is widely used in traditional Chinese medicine researches to discover bioactive components and analyze drug action mechanisms by detecting the relationship between each drug component and its target proteins.^[17,19]^ Despite significant research efforts, the exact mechanisms underlying the therapeutic effects of TWH for BD treatment remain unknown. Therefore, we employed network pharmacology and molecular docking to explore the impact of TWH on BD, hoping that our findings will provide additional evidence and guidance for the treatment of BD. As shown in Figure 1, we identified the active ingredients and core targets of TWH for BD treatment and verified their potential relationship through molecular docking.

## 2. Materials and methods

### 2.1. Obtaining TWH ingredients and therapeutic targets

The Traditional Chinese medicine systems pharmacology database and analysis platform^[20]^ (TCMSP, https://old.tcmsp-e.com/tcmsp.php) was used to screen for active ingredients and potential therapeutic targets of TWH. We used oral relative bioavailability (OB), which reflects the ability of compounds to reach systemic circulation, and drug-likeness (DL), which assesses the possibility of a molecule becoming an oral drug with respect to factors such as bioavailability, to identify the potential bioactive compounds of TWH. Ingredients with OB ≥ 30%, and DL ≥ 0.18 were considered as candidate active compounds as previously reported.^[21]^ The corresponding genes of these bioactive ingredients were identified as potential therapeutic targets. Ethical approval is not required for this study as it pertains to network pharmacology and bioinformatics, and does not involve any ethical concerns related to clinical or animal experiments.

### 2.2. Acquisition of BD target genes

The acquisition of BD target genes was performed via 2 databsases, DisGeNet (https://www.disgenet.org/) and GeneCards (https://www.genecards.org/), with the search term “Behcet disease” or “Behcet syndrome.” The harvested BD targets and TWH targets were visualized by Venn diagram to determine intersection targets using the R package VennDiagram (v1.7.1). The corresponding active compounds of the intersection targets were also identified.

### 2.3. Construction of TWH–ingredients–gene–BD network

A network of complex information based on interactions among TWH, bioactive ingredients of TWH, gene symbols of BD targets, and BD was constructed using Cytoscape (v3.9.0). Each node in the network represents TWH, BD, a bioactive compound of TWH, or a target gene of BD. Each edge is a representation of the relationship between each node within the network.

### 2.4. Construction of a protein-protein interaction (PPI) network and identification of core targets

Gene symbols of the BD targets were converted into UniProt identifiers using the R package AnnotationDbi (v1.52.0). A PPI network was constructed by inputting the UniProt identifiers of BD targets into the STRING network platform (https://string-db.org) with default parameters. To identify the core targets of TWH, the network information was downloaded from the STRING platform and imported into Cytoscape. Target proteins in the network were ranked by the maximal clique centrality (MCC) algorithm using the Cytohubba plug-in-in.^[22]^ The top 6 targets were selected as the core target proteins.

### 2.5. Gene ontology (GO) annotation and Kyoto encyclopedia of genes and genomes (KEGG) enrichment analysis of intersection genes

We performed GO annotation and KEGG pathway enrichment analysis to understand the functions of the intersection genes. GO annotation provides systematic functional properties of genes in 3 categories: biological processes, molecular functions and cellular components. Additionally, KEGG pathway enrichment uncovers the metabolic pathways of genes and the functions of the gene products. Enrichment analysis was performed in R (v4.0.2) using the org.Hs.e.g..db (v3.12.0) and clusterProfiler (v3.18.0) packages. The parameters for enrichment analysis were set as follows: *P* value cutoff = .05, *P* value adjust method = “BH” (Benjamini-Hochberg procedure), *Q* value cutoff = 0.2. The top 5 elements of each GO category and the top 10 enriched KEGG pathways were visualized in bubble plots using the R package ggplot (v3.3.5). In the bubble plot, each bubble size indicates the number of enriched targets and the color of bubble represents the BH-adjusted *P* value of enrichment.

### 2.6. Molecular docking between core target protein and bioactive TWH ingredient

We used molecular docking technique to investigate the intermolecular interaction between the core target proteins and bioactive TWH ingredients in a 3-dimensional space. The top 6 core target proteins and their corresponding bioactive TWH ingredients were introduced into the molecular docking process. The 3D structures of the target proteins were obtained from the RCSB PDB (http://www.rcsb.org/) in PDB format. The structures of TWH bioactive ingredients were retrieved from the TCMSP database in mol2 format. We employed the cavity-detection guided blind docking (CB-Dock) algorithm to perform molecular docking. CB-Dock^[23]^ utilizes a curvature-based cavity-detection approach to predict binding sites, calculate the centers and sizes of a possible ligand binding cavity and then performs molecular docking with AutoDock Vina to search for an optimal conformation. CB-Dock performs blind docking at the predicted sites instead of the entire protein surface, making it more efficient than other blind docking tools.^[23]^ The stand-alone version of CB-Dock (available at http://clab.labshare.cn/cb-dock/php/manual.php) was used in this study with the following parameters: number of cavities for docking = 5, AutoDock Vina exhaustiveness = 32. Binding affinity was used to evaluate the binding capacity of the protein and the bioactive TWH ingredient. A greater negative binding affinity value corresponds to a higher stability of the bioactive ingredient-protein complex. Detailed molecular docking information was further analyzed using Discovery Studio Visualizer (v21.1.0).

## 3. Results

### 3.1. Screening of bioactive ingredients and corresponding targets of TWH in treatment of BD

**Figure 2.**
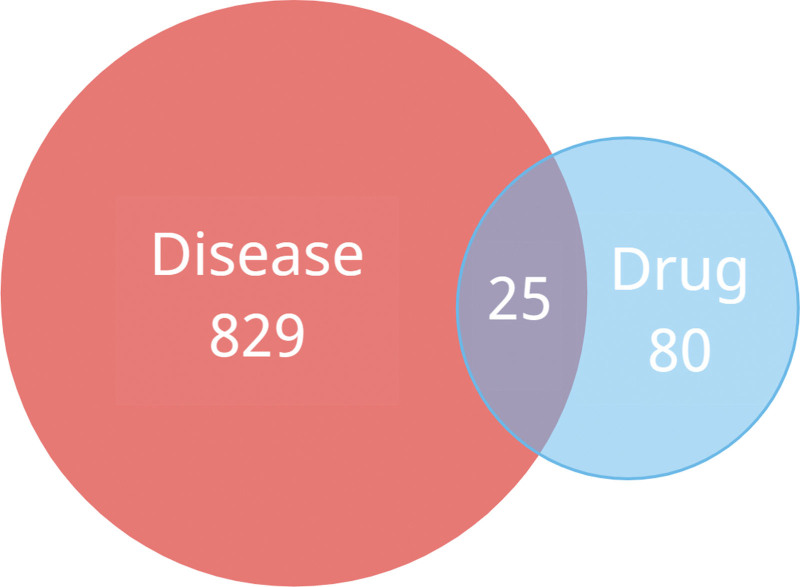
Venn gram of BD related target genes (disease) and TWH-related target genes (drug). BD = Behcet’s disease, TWH = *Tripterygium wilfordii* hook.

**Figure 3.**
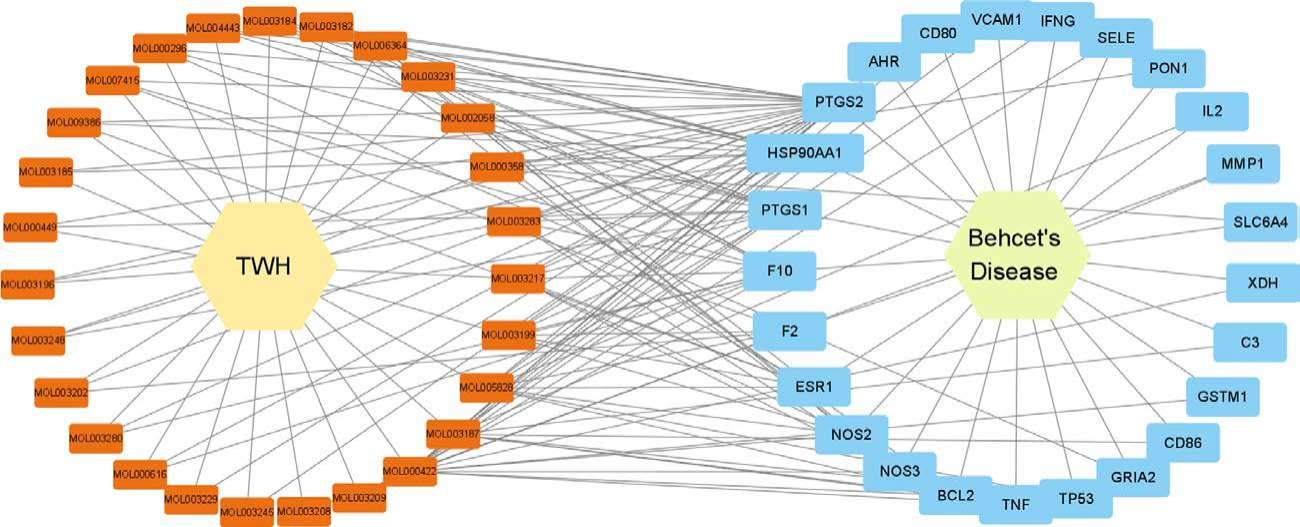
TWH–Ingredients–Gene–BD network. Each node in the network represents TWH, BD, a bioactive compound of TWH, or a BD target gene. Each edge of the network is a representation of the relationship between each node. BD = Behcet’s disease, TWH = *Tripterygium wilfordii* hook.

From the 144 TWH ingredients recorded in TCMSP, 58 bioactive ingredients were selected based on their OB and DL values, as presented in Table 1, and 105 corresponding target genes (See Table S1, Supplemental Digital Content, http://links.lww.com/MD/K482, which demonstrates TWH and BD related genes and intersection of TWH and BD related genes) were identified as TWH therapeutic targets. In addition, 502 and 658 potential targets were retrieved from DisGeNet and GeneCards, respectively (See Table S1, Supplemental Digital Content, http://links.lww.com/MD/K482, which demonstrates TWH and BD related genes and intersection of TWH and BD related genes). After the removal of duplicated targets, the final set of BD targets consisted of 854 genes (See Table S1, Supplemental Digital Content, http://links.lww.com/MD/K482, which demonstrates TWH and BD related genes and intersection of TWH and BD related genes). The TWH and BD target genes were visualized using a Venn diagram, and 25 intersection genes related to both TWH and BD were acquired (Fig. 2 and Table S1, Supplemental Digital Content, http://links.lww.com/MD/K482, which demonstrates TWH and BD related genes and intersection of TWH and BD related genes). Furthermore, 31 TWH ingredients whose target genes were not included in the intersection targets were excluded from the list of potential bioactive TWH ingredients. Finally, a TWH–Ingredients–Gene–BD network was constructed with 27 active ingredients and 25 corresponding targets (Table 2, Fig. 3). By analyzing the degrees of each ingredient node, kaempferol (MOL000422) with 14 edges was found to be the most important active ingredient of TWH. Similarly, prostaglandin-endoperoxide synthase 2 (PTGS2) with 25 edges was identified as a potential key gene target for BD treatment.

**Table 1:** Bioactive ingredients of *Tripterygium wilfordii* hook with relative bioavailability (OB) ≥ 30% and drug-likeness (DL) ≥ 0.18.

Molecule ID	Molecule name	OB (%)	DL	MW
MOL000296	Hederagenin	36.91	0.75	414.79
MOL003181	(2S,3R,4S,5S,6R)-2-[4-[(1S,3aR,4S,6aR)-4-[3-methoxy-4-[(2S,3R,4S,5S,6R)-3,4,5-trihydroxy-6-(hydroxymethyl) oxan-2-yl] oxyphenyl]-1,3,3a,4,6,6a-hexahydrofuro[4,3-c] furan-1-yl]-2,6-dimethoxyphenoxy]-6-(hydroxymethyl) oxane-3,4,5-triol	9.05	0.32	712.77
MOL003182	(+)-Medioresinol di-O-beta-D-glucopyranoside_qt	60.69	0.62	388.45
MOL003184	81827-74-9	45.42	0.53	342.47
MOL003185	(1R,4aR,10aS)-5-hydroxy-1-(hydroxymethyl)-7-isopropyl-8-methoxy-1,4a-dimethyl-4,9,10,10a-tetrahydro-3H-phenanthren-2-one	48.84	0.38	346.51
MOL003187	Triptolide	51.29	0.68	360.44
MOL003188	Tripchlorolide	78.72	0.72	396.9
MOL003189	Wilforlide A	35.66	0.72	486.81
MOL003192	Triptonide	67.66	0.7	344.39
MOL003196	Tryptophenolide	48.5	0.44	312.44
MOL003198	5 alpha-Benzoyl-4 alpha-hydroxy-1 beta,8 alpha-dinicotinoyl-dihydro-agarofuran	35.26	0.72	600.72
MOL003199	5,8-Dihydroxy-7-(4-hydroxy-5-methyl-coumarin-3)-coumarin	61.85	0.54	352.31
MOL003202	8-epi-Loganic acid	4.43	0.4	376.4
MOL003206	Canin	77.41	0.33	278.33
MOL003208	Celafurine	72.94	0.44	369.51
MOL003209	Celallocinnine	83.47	0.59	405.59
MOL003210	Celapanine	30.18	0.82	569.66
MOL003211	Celaxanthin	47.37	0.58	550.94
MOL003217	Isoxanthohumol	56.81	0.39	354.43
MOL003222	Salazinic acid	36.34	0.76	402.33
MOL003224	Tripdiotolnide	56.4	0.67	360.44
MOL003225	Hypodiolide A	76.13	0.49	318.5
MOL003229	Triptinin B	34.73	0.32	314.46
MOL003231	Triptoditerpenic acid B	40.02	0.36	328.49
MOL003232	Triptofordin B1	39.55	0.84	478.63
MOL003234	Triptofordin C2	30.16	0.76	610.71
MOL003235	Triptofordin D1	32	0.75	606.72
MOL003236	Triptofordin D2	30.38	0.69	650.78
MOL003237	(3E,7E)-2alpha,10beta,13alpha-Triacetoxy-5alpha,20-dihydroxy-3,8-seco-taxa-3,7,11-trien-9-one	7.93	0.69	492.62
MOL003238	Triptofordin F1	33.91	0.6	694.79
MOL003239	Triptofordin F2	33.62	0.67	668.75
MOL003240	Triptofordin F3	8.04	0.6	710.79
MOL003241	Triptofordin F4	31.37	0.67	652.75
MOL003242	Triptofordinine A2	30.78	0.47	741.85
MOL003244	Triptonide	68.45	0.68	358.42
MOL003245	Triptonoditerpenic acid	42.56	0.39	344.49
MOL003248	Triptonoterpene	48.57	0.28	300.48
MOL003249	Triptoriterpenic acid A	9.69	0.71	488.78
MOL003266	21-Hydroxy-30-norhopan-22-one	34.11	0.77	428.77
MOL003267	Wilformine	46.32	0.2	805.86
MOL003273	Euonine	7.5	0.2	805.86
MOL003278	Salaspermic acid	32.19	0.63	472.78
MOL003279	99694-86-7	75.23	0.66	376.44
MOL003280	Triptonolide	49.51	0.49	326.42
MOL000358	Beta-sitosterol	36.91	0.75	414.79
MOL000616	(+)-Suyringaresinol-di-O-beta-D-glucoside	5.19	0.29	742.8
MOL000211	Mairin	55.38	0.78	456.78
MOL000422	Kaempferol	41.88	0.24	286.25
MOL000449	Stigmasterol	43.83	0.76	412.77
MOL002058	40957-99-1	57.2	0.62	388.45
MOL003283	(2R,3R,4S)-4-(4-hydroxy-3-methoxy-phenyl)-7-methoxy-2,3-dimethylol-tetralin-6-ol	66.51	0.39	360.44
MOL004443	Zhebeiresinol	58.72	0.19	280.3
MOL005828	Nobiletin	61.67	0.52	402.43
MOL006364	(2R,3R,4S)-4-(4-hydroxy-3,5-dimethoxy-phenyl)-5,7-dimethoxy-2,3-dimethylol-tetralin-6-ol	4.87	0.54	420.5
MOL007415	[(2S)-2-[[(2S)-2-(benzoylamino)-3-phenylpropanoyl] amino]-3-phenylpropyl] acetate	58.02	0.52	444.57
MOL007535	(5S,8S,9S,10R,13R,14S,17R)-17-[(1R,4R)-4-ethyl-1,5-dimethylhexyl]-10,13-dimethyl-2,4,5,7,8,9,11,12,14,15,16,17-dodecahydro-1H-cyclopenta[a]phenanthrene-3,6-dione	33.12	0.79	428.77
MOL009386	3,3’-bis-(3,4-dihydro-4-hydroxy-6-methoxy)-2H-1-benzopyran	52.11	0.54	358.42
MOL011169	Peroxyergosterol	44.39	0.82	428.72

OB = oral relative bioavailability; DL = drug-likeness; MW = molecular weight.

**Table 2:** Bioactive ingredients of *Tripterygium wilfordii* hook and their target BD related genes.

Molecule ID	Molecule name	Target genes (BD related)
MOL000296	Hederagenin	*GRIA2/PTGS1/PTGS2*
MOL000358	Beta-sitosterol	*BCL2/HSP90AA1/PON1/PTGS1/PTGS2/SLC6A4*
MOL000422	Kaempferol	*AHR/BCL2/F2/GSTM1/HSP90AA1/MMP1/NOS2/NOS3/PTGS1/PTGS2/SELE/TNF/VCAM-1/XDH*
MOL000449	Stigmasterol	*PTGS1/PTGS2*
MOL000616	(+)-Suyringaresinol-di-O-beta-D-glucoside	*F10/PTGS2*
MOL002058	40957-99-1	*F10/HSP90AA1/NOS3/PTGS1/PTGS2*
MOL003182	(+)-Medioresinol di-O-beta-D-glucopyranoside_qt	*F10/HSP90AA1/NOS3/PTGS2*
MOL003184	81827-74-9	*F10/HSP90AA1/PTGS1/PTGS2*
MOL003185	(1R,4aR,10aS)-5-hydroxy-1-(hydroxymethyl)-7-isopropyl-8-methoxy-1,4a-dimethyl-4,9,10,10a-tetrahydro-3H-phenanthren-2-one	*HSP90AA1/PTGS2*
MOL003187	Triptolide	*BCL2/C3/CD80/CD86/IFNG/IL-2/PTGS2/TNF/TP53*
MOL003196	Tryptophenolide	*HSP90AA1/PTGS2*
MOL003199	5,8-Dihydroxy-7-(4-hydroxy-5-methyl-coumarin-3)-coumarin	*ESR1/F10/F2/HSP90AA1/NOS2/PTGS1/PTGS2*
MOL003202	8-epi-Loganic acid	*F2/PTGS2*
MOL003208	Celafurine	*F2*
MOL003209	Celallocinnine	*F10*
MOL003217	Isoxanthohumol	*ESR1/F10/HSP90AA1/NOS2/NOS3/PTGS1/PTGS2*
MOL003229	Triptinin B	*PTGS2*
MOL003231	Triptoditerpenic acid B	*F10/HSP90AA1/PTGS1/PTGS2*
MOL003245	Triptonoditerpenic acid	*PTGS2*
MOL003248	Triptonoterpene	*PTGS1/PTGS2*
MOL003280	Triptonolide	*F10/PTGS2*
MOL003283	(2R,3R,4S)-4-(4-hydroxy-3-methoxy-phenyl)-7-methoxy-2,3-dimethylol-tetralin-6-ol	*ESR1/F10/HSP90AA1/NOS3/PTGS1/PTGS2*
MOL004443	Zhebeiresinol	*HSP90AA1/PTGS1/PTGS2*
MOL005828	Nobiletin	*BCL2/ESR1/F10/F2/HSP90AA1/NOS2/PTGS1/PTGS2/TP53*
MOL006364	(2R,3R,4S)-4-(4-hydroxy-3,5-dimethoxy-phenyl)-5,7-dimethoxy-2,3-dimethylol-tetralin-6-ol	*ESR1/HSP90AA1/NOS2/PTGS2*
MOL007415	[(2S)-2-[[(2S)-2-(benzoylamino)-3-phenylpropanoyl] amino]-3-phenylpropyl] acetate	*F10/F2/PTGS2*
MOL009386	3,3’-bis-(3,4-dihydro-4-hydroxy-6-methoxy)-2H-1-benzopyran	*ESR1/HSP90AA1/PTGS2*

BD = Behcet’s disease, PTGS2 = prostaglandin-endoperoxide synthase 2.

### 3.2. Construction of PPI network and identification of core targets

**Figure 4.**
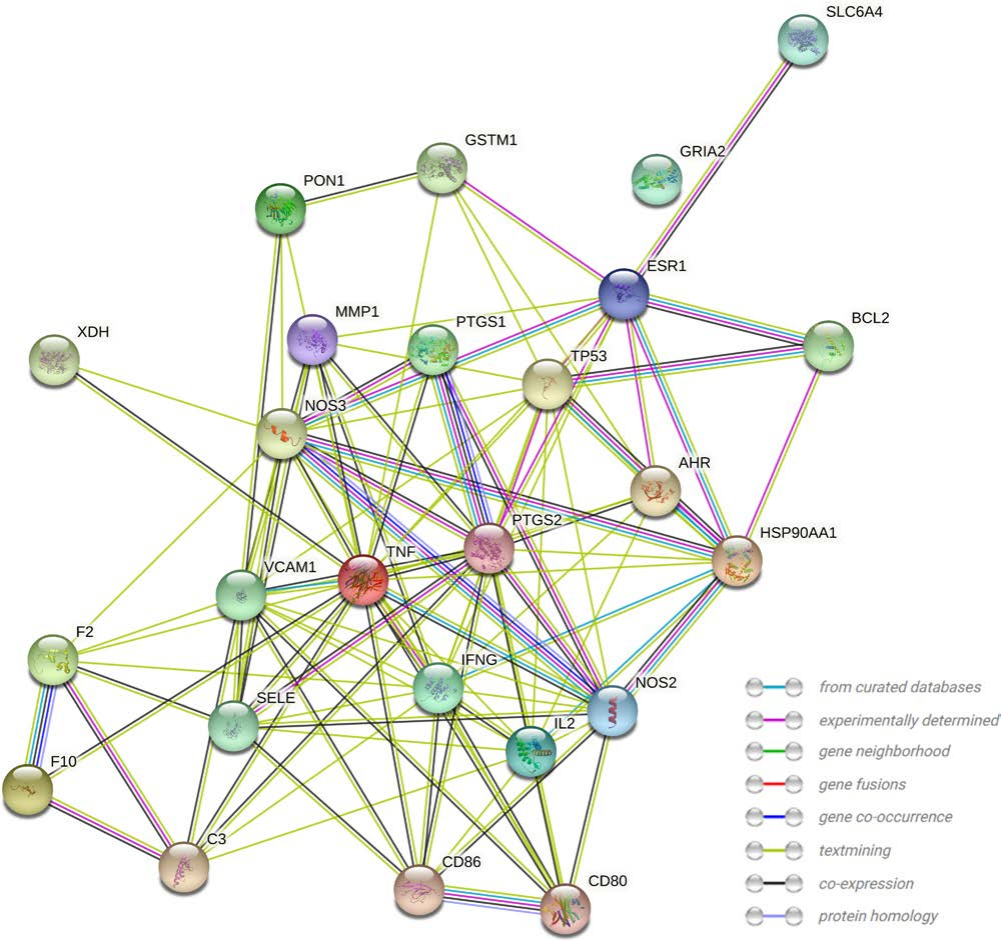
Protein-protein interaction (PPI) network of BD target proteins. BD = Behcet’s disease.

**Figure 5.**
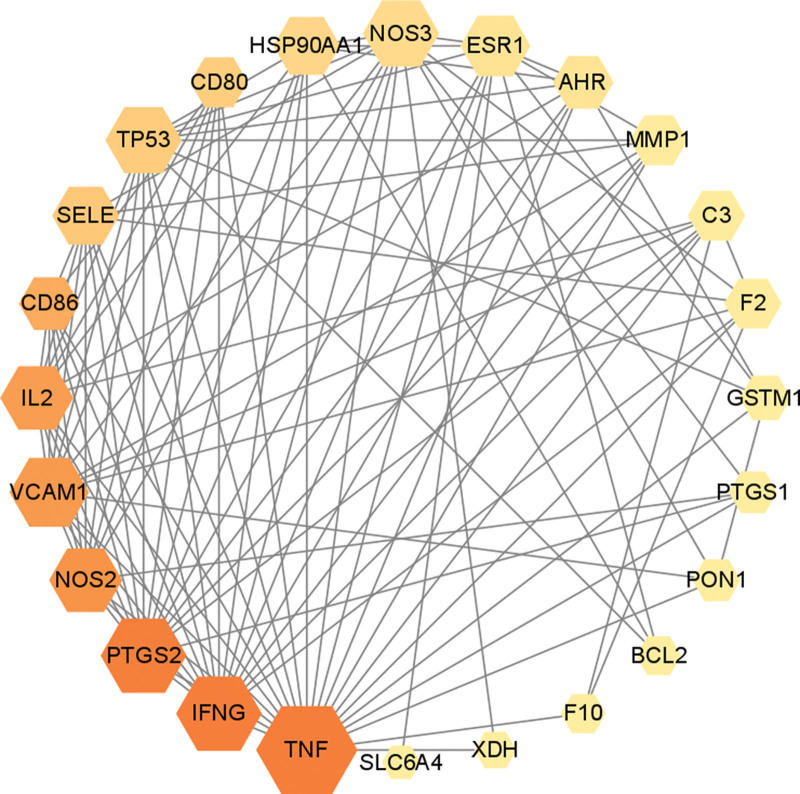
Identification of core targets of TWH in treatment BD. Each node in the network represents a target protein. The color and size indicate the maximal clique centrality (MCC) and degree of each node. BD = Behcet’s disease, TWH = *Tripterygium wilfordii* hook.

To further investigate the function of the 25 intersection genes, we constructed a PPI network by inputting these targets into the STRING network platform (Fig. 4). The resulting PPI network had 25 nodes with 107 edges and its degree was found significantly higher than the expected number of edges (107 vs 32, *P* < .01), indicating a biologically connected network. The PPI network data were then imported into Cytoscape, and the targets were analyzed by ordering the MCC score of each target using the Cytohubba plug-in. As shown in Table 3 and Figure 5, TNF, IFNG, PTGS2, NOS2, VCAM-1, and interleukin-2 (IL-2) got the highest MCC scores and were thus regarded as the core targets of TWH for BD treatment.

**Table 3:** Top six gene targets in protein-protein network.

No	Gene symbol	Gene name	MCC	Degree
1	*TNF*	Tumor necrosis factor	16,528	14
2	*IFNG*	Interferon gamma	16,464	14
3	*PTGS2*	Prostaglandin G/H synthase 2	16,344	14
4	*NOS2*	Nitric oxide synthase, inducible	13,704	11
5	*VCAM-1*	Vascular cell adhesion protein 1	12,750	11
6	*IL-2*	Interleukin-2	12,360	11

IL-2 = interleukin-2, MCC = maximal clique centrality, PTGS2 = prostaglandin-endoperoxide synthase 2.

### 3.3. GO annotation and KEGG enrichment analysis of intersection genes

**Figure 6.**
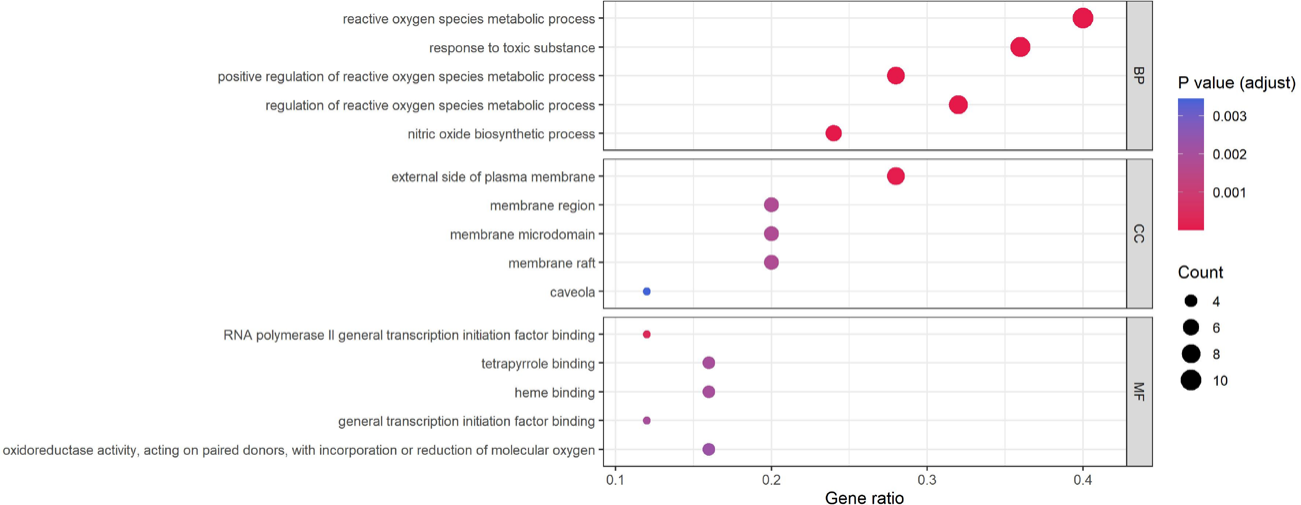
GO annotation analysis of intersection genes. GO = gene ontology.

**Figure 7.**
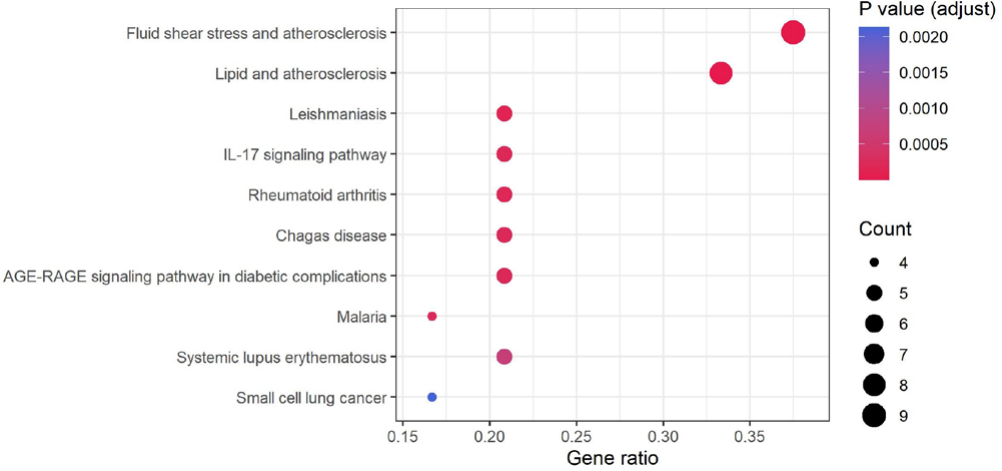
KEGG enrichment analysis of intersection genes. KEGG = Kyoto encyclopedia of genes and genomes.

GO annotation and KEGG enrichment analysis were conducted to elucidate the function of the 25 target genes. As presented in Figure 6, several biological processes were found to be involved, and the most significantly enriched biological processes were reactive oxygen species metabolic process, response to toxic substance and positive regulation of reactive oxygen species metabolic process. Most targets were located on the external side of the plasma membrane. The top-ranked molecular functions were RNA polymerase II general transcription initiation factor binding, tetrapyrrole binding, and heme binding. These results suggest that TWH may be involved in the treatment of BD through a variety of pathways in which the manipulation of reactive oxygen species may play an important role. Moreover, KEGG analysis identified 65 enriched signaling pathways. As shown in Figure 7, fluid shear stress and atherosclerosis were the most significantly enriched pathways. Detailed information on GO annotation and KEGG enrichment analysis is available in Table S2, Supplemental Digital Content, http://links.lww.com/MD/K483, which demonstrates detailed information of GO annotation result) and Table S3, Supplemental Digital Content, http://links.lww.com/MD/K484, which demonstrates detailed information of KEGG enrichment analysis results).

### 3.4. Validation of TWH ingredient in treatment of BD by molecular docking

**Figure 8.**
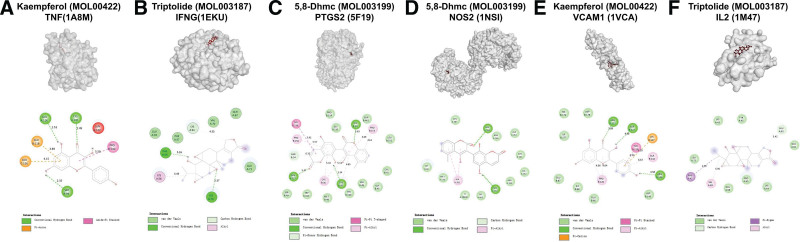
Molecular docking of BD related targets with bioactive ingredients of TWH. (A) Interaction between kaempferol (MOL00422) and TNF (18AM), (B) interaction between triptolide (MOL003187) and IFNG(1EKU), (C) interaction between 5, 8-Dhmc (MOL003199) and PTGS2 (5F19), (D) interaction between 5, 8-Dhmc (MOL003199) and NOS2 (1NSI), (E) interaction between kaempferol (MOL00422) and VCAM-1 (1VCA), and (F) interaction between triptolide (MOL003187) and IL-2 (1M47). BD = Behcet’s disease, IL-2 = interleukin-2, PTGS2 = prostaglandin-endoperoxide synthase 2, TWH = *Tripterygium wilfordii* hook.

According to the previous analysis, 25 bioactive ingredients of TWH were found to bind to the core targets (TNF, IFNG, PTGS2, NOS2, VCAM-1, and IL-2). Molecular docking analysis was performed to explore the binding capacity between the bioactive ingredients and their corresponding targets (Table 4). Among all the molecular docking analysis, kaempferol (MOL00422) showed the highest binding affinity of −8.25 kcal/mol with 1A8M (TNF) and it could also bind to 1VCA (VCAM-1) with an affinity of −7.13 kcal/mol. Kaempferol bound to IA8M (TNF) via 3 conventional hydrogen bonds with GLU-A116, CYS-A101, and SER-B99. Other forces including pi-anion and anion-pi stacked were also investigated (Fig. 8A). Three conventional hydrogen bonds together with van der Waals, pi-pi stacked, pi-alkyl, pi-cation interactions were found between kaempferol and 1VCA (VCAM-1) (Fig. 8E). Among all the molecules analyzed, triptolide (MOL003187) could bind to 1EKU (IFNG) and 1M47 (IL-2) with the highest binding affinity of −6.851 kcal/mol and −6.255 kcal/mol, respectively. Triptolide was attracted to 1EKU (IFNG) by 1 hydrogen bond, an alkyl, a carbon hydrogen bond and van der Waals forces (Fig. 8B). Triptolide could also formed van der Waals forces, pi-sigma, alkyl and carbon hydrogen bond with 1M47 (IL-2) (Fig. 8F). Furthermore, 5,8-Dihydroxy-7-(4-hydroxy-5-methyl-coumarin-3)-coumarin (MOL003199), abbreviated as 5, 8-Dhmc, showed the highest affinity of −11.02 kcal/mol with 5F19 (PTGS2) via 3 hydrogen bonds, pi-pi T-shaped, pi-alkyl, pi-donor hydrogen bond and van der Waals forces (Fig. 8C). 5, 8-Dhmc could also bind to NOS2 (1NSI) with an affinity of −8.26 kcal/mol via 2 hydrogen bonds, pi-pi T-shaped, pi-alkyl, pi-donor hydrogen bond and van der Waals forces (Fig. 8D). Detailed molecular docking results are available in Supplemental Digital Content 1, http://links.lww.com/MD/K485, which provides detailed information on molecular docking results).

**Table 4:** Molecular docking for bioactive molecular of *Tripterygium wilfordii* hook and their corresponding target proteins. For each target protein, molecule with the highest binding affinity is bolded.

Targets (PDB ID)	Molecular ID	Binding affinity (kcal/mol)	Center*			Size*		
			x	y	z	x	y	z
TNF (1A8M)	MOL000422 (Kaempferol)	−8.25	8.59	62.48	31.14	21	21	21
	MOL003187	−6.82	8.59	62.48	31.14	21	21	21
IFNG (1EKU)	MOL003187 (Triptolide)	−6.85	7.32	35.43	22.60	31	21	35
PTGS2 (5F19)	MOL003199 (5,8-Dhmc)	−11.02	10.63	32.08	25.00	33	35	28
	MOL003217	−9.80	10.63	32.08	25.00	33	35	28
	MOL003182	−9.58	10.63	32.08	25.00	33	35	23
	MOL003196	−9.48	10.63	32.08	25.00	33	35	28
	MOL000422	−9.43	34.04	50.33	55.01	35	30	29
	MOL009386	−9.38	10.63	32.08	25.00	33	35	23
	MOL002058	−9.34	10.63	32.08	25.00	33	35	24
	MOL003229	−9.23	10.63	32.08	25.00	33	35	28
	MOL005828	−9.07	10.63	32.08	25.00	33	35	23
	MOL000616	−9.00	34.04	50.33	55.01	31	31	31
	MOL003184	−8.90	10.63	32.08	25.00	33	35	28
	MOL007415	−8.85	34.04	50.33	55.01	35	30	24
	MOL003187	−8.83	34.04	50.33	55.01	35	30	29
	MOL000449	−8.81	10.63	32.08	25.00	33	35	25
	MOL003280	−8.73	34.04	50.33	55.01	35	30	29
	MOL000296	−8.54	10.63	32.08	25.00	33	35	25
	MOL003245	−8.51	10.63	32.08	25.00	33	35	28
	MOL003231	−8.43	13.10	48.31	64.50	20	28	29
	MOL003202	−8.31	10.63	32.08	25.00	33	35	28
	MOL003185	−8.27	10.63	32.08	25.00	33	35	28
	MOL000358	−8.20	10.63	32.08	25.00	33	35	25
	MOL003248	−8.02	10.63	32.08	25.00	33	35	28
	MOL004443	−7.96	10.63	32.08	25.00	33	35	28
	MOL006364	−7.82	34.04	50.33	55.01	35	30	29
	MOL003283	−7.78	10.63	32.08	25.00	33	35	28
NOS2 (1NSI)	MOL003199 (5,8-Dhmc)	−8.26	14.23	59.18	28.69	35	28	35
	MOL000422	−7.88	14.23	59.18	28.69	35	28	35
	MOL003217	−7.62	14.23	59.18	28.69	35	28	35
	MOL006364	−7.16	65.23	17.52	66.44	21	21	21
	MOL005828	−6.85	65.23	17.52	66.44	23	23	23
VCAM1 (1VCA)	MOL000422 (Kaempferol)	−7.13	10.97	11.83	72.09	21	21	21
IL2 (1M47)	MOL003187 (Triptolide)	−6.26	17.36	22.67	19.35	21	21	21

Molecular name: MOL000296: Hederagenin; MOL000358: beta-sitosterol; MOL000422: Kaempferol; MOL000449: Stigmasterol; MOL000616: (+)-Suyringaresinol-di-O-beta-D-glucoside; MOL002058: 40957-99-1; MOL003182: (+)-Medioresinol di-O-beta-D-glucopyranoside_qt; MOL003184: 81827-74-9; MOL003185: (1R,4aR,10aS)-5-hydroxy-1-(hydroxymethyl)-7-isopropyl-8-methoxy-1,4a-dimethyl-4,9,10,10a-tetrahydro-3H-phenanthren-2-one; MOL003187: Triptolide; MOL003196: Tryptophenolide; MOL003199: 5,8-Dihydroxy-7-(4-hydroxy-5-methyl-coumarin-3)-coumarin (Synonym: 5,8-Dhmc); MOL003202: 8-epi-Loganic acid; MOL003217: Isoxanthohumol; MOL003229: Triptinin B; MOL003231: Triptoditerpenic acid B; MOL003245: Triptonoditerpenic acid; MOL003248: Triptonoterpene; MOL003280: Triptonolide; MOL003283: (2R,3R,4S)-4-(4-hydroxy-3-methoxy-phenyl)-7-methoxy-2,3-dimethylol-tetralin-6-ol; MOL004443: Zhebeiresinol; MOL005828: Nobiletin; MOL006364: (2R,3R,4S)-4-(4-hydroxy-3,5-dimethoxy-phenyl)-5,7-dimethoxy-2,3-dimethylol-tetralin-6-ol; MOL007415: [(2S)-2-[[(2S)-2-(benzoylamino)-3-phenylpropanoyl] amino]-3-phenylpropyl] acetate; MOL009386: 3,3’-bis-(3,4-dihydro-4-hydroxy-6-methoxy)-2H-1-benzopyran.

PTGS2 = prostaglandin-endoperoxide synthase 2.

* The center and dimensions (in Angstrom) of the docking box.

## 4. Discussion

BD is a rare autoimmune disease, characterized by a wide range of clinical manifestations and alternating periods of recurrence and remission. Although many genetic, environmental, and immunological factors have been identified for the development of BD, the pathogenesis of BD remains poorly understood.^[24]^ In Chinese traditional medicine, TWH has been found to have significant anti-inflammatory properties and is widely used in various immune-mediated inflammatory diseases.^[25]^ Although several studies have elucidated the mechanism of TWH in the treatment of rheumatoid arthritis^[26]^ and ankylosing spondylitis,^[27]^ few have investigated the mechanism of TWH in BD treatment. In our study, we explored the mechanism of TWH in BD treatment using network pharmacology and molecular docking.

The role of TNF-α in the pathogenesis of BD has been emphasized by numerous studies which reported elevated levels of TNF-α in the serum of patients with BD. Furthermore, TNF-α concentration was found positively correlated with BD activity in a cross-sectional, blinded study.^[28]^ Therapeutic administration of TNF-α blockers has been successfully used to treat various manifestations of BD.^[29]^ TNF-α exerts pro-inflammatory effects via binding to tumor necrosis factor receptor which activates the NF-κB pathway.^[30]^ In our study, TNF ranked first among the core targets, indicating its critical role in TWH treatment for BD. This result is consistent with a cohort study which found that serum levels of TNF-α in BD patients were significantly lowered after 3-month of Tripterygium glycosides treatment.^[14]^ In addition, molecular docking analysis confirmed the interaction between triptolide, a TWH ingredient, and TNF. Although previous researches reported that triptolide could inhibit the function of TNF-α in osteoblast differentiation^[31]^ and mRNA expression levels of TNF-α in ankylosing spondylitis rat model,^[32]^ further research is required to provide evidence for clinical effectiveness and mechanism of triptolide in BD.

IFN-γ and IL-2, primarily produced by antigen-activated T cells, are immunoregulatory cytokines.^[33]^ According to a cross-sectional study, significantly elevated serum levels of IFN-γ were detected in patients with active stage of BD compared to patients with inactive BD or controls.^[34]^ In another cross-sectional study, Alposy et al found that plasma level of IL-2 was increased in patients with active BD and soluble interleukin-2 receptor (sIL-2R) levels in patients with active BD were significantly higher than those in patients with inactive BD or controls.^[35]^ Similarly, in a cross-sectional study by Sugi-Ikai et al, it was discovered that active BD patients had significantly higher frequencies of IL-2- and IFN-γ producing CD4 + and CD8 + cells in comparison to inactive BD patients.^[36]^ Additionally, a significantly increased frequency of IL-2 (−330) GG genotype was observed in patients with BD.^[37]^ These results provide evidence that IFN-γ and IL-2 may play a role in the pathogenesis of BD. Furthermore, cellular studies revealed that triptolide, which had high binding ability to IFN-γ and IL-2 according to our molecular docking analysis, could inhibit IFN-γ signal transduction^[38]^ and T cell IL-2 production.^[39]^ Thus, the regulation of inflammatory cytokines may play a significant role in bioactive ingredients of TWH against BD.

PTGS2, also known as cyclooxygenase 2 (COX2), is an inducible enzyme which is involved in prostaglandin biosynthesis during the immune response to inflammation.^[40]^ COX2 activity was found to be significantly increased in neutrophils obtained from patients with BD^[41]^ and nonsteroidal anti-inflammatory drugs (NSAIDS), which are COX inhibitors, are commonly used to treat joint pains in patients with BD. In our study, we identified 25 potential ingredients of TWH, including triptolide, which can interact with COX2. Consistent with our molecular docking analysis, Geng et al found that triptolide directly inhibited the expression of both COX2 mRNA and protein, thereby reducing prostaglandin synthesis.^[42]^ Among all the bioactive compounds of TWH analyzed, the binding affinity between 5,8-Dhmc and COX2 was the highest. Further study is required to clarify the possibility of 5,8-Dhmc in alleviating the symptoms related to BD.

VCAM-1 is a cell adhesion molecule which participates in the recruitment of leukocytes to endothelial cells and is closely associated with various immunological disorders.^[43]^ Chronic inflammation in BD leads to endothelial cell damage and activates endothelial cells via the release of various pro-inflammatory cytokines which upregulates endothelial cell expression of adhesion molecules (VCAM-1).^[44]^ A cross-sectional clinical study found that the serum levels of VCAM-1 were significantly higher in patients with BD than in controls.^[45]^ In addition, a cohort study by Yang et al^[16]^ reported that the expression of sVCAM-1, a soluble form of VCAM-1, decreased significantly after a 2-month treatment with TWH. Therefore, VCAM-1 may be a potential therapeutic target for BD. We predicted that VCAM-1 may be affected by kaempferol via molecular docking. Surprisingly, this result is consistent with previous studies. In a study of kaempferol against inflammatory bowel disease, kaempferol significantly reduced the overproduction of VCAM-1 induced by lipopolysaccharide,^[46]^ indicating the potential role of kaempferol in the regulation of VCAM-1.

According to GO annotation, the metabolic process of reactive oxygen species (ROS) plays an essential role in TWH in the treatment of BD. ROS are highly active metabolites of oxygen with strong oxidizing capabilities and serve as signaling molecules in the progression of inflammatory disorders.^[47]^ At the site of inflammation, ROS generated by neutrophils causes endothelial dysfunction, which promotes the migration of inflammatory cells, leading to aggravated inflammation and tissue injury.^[47]^ Several studies have revealed that ROS, mainly produced by neutrophils, are involved in the pathogenesis of BD. Serum ROS levels were higher in patients with BD than in healthy controls, and neutrophils from patients with BD produced more ROS.^[48]^ According to a cross-sectional study, enhanced activities of ROS-producing enzymes such as NADPH oxidase, myeloperoxidase and COX2 have been detected in patients with BD.^[41]^ Moreover, nitric oxide, another important source of oxidative stress, was also found to be increased in BD patients.^[16,49]^ NOS2, also named inducible nitric oxide synthase (iNOS), was identified as a core target in our study. During inflammation, cytokines like TNF-α and IFN-γ trigger the induction of iNOS, which in turn boosts the production of nitric oxide.^[50]^ Furthermore, a reduction in antioxidant defense enzymes, particularly superoxide dismutase and glutathione peroxidase, was identified in patients with BD based on a cross-sectional study.^[51]^ Our study revealed that the fluid shear stress and atherosclerosis pathways were the most significantly enriched pathways according to KEGG analysis. This pathway has been implicated as a key process involving ROS,^[52]^ further emphasizing the importance of antioxidants in the treatment of BD. Moreover, a systemic review by Mira et al demonstrated that subclinical atherosclerosis is prevalent in BD, as indicated by lower flow-mediated dilatation and endothelial-mediated dilatation than in controls, and greater intima-media thickness of the carotid arteries in patients with BD.^[53]^ Thus, antioxidation may be a potential therapeutic target for BD.

Several ingredients of TWH have been reported to possess antioxidant capacities. In a rat model, Tripterygium glycoside inhibited oxidative stress and enhanced antioxidative ability by increasing the activities of catalase and glutathione peroxidase.^[54]^ Triptolide was found to inhibit the level of local and systemic oxidative stress by decreasing the activity of myeloperoxidase and iNOS.^[55]^ Additionally, Kaempferol is a potent ROS scavenger which modulates the expression of heme oxygenase-1 and the mitogen-activated protein kinase pathway.^[56,57]^ IFN-γ, TNF, COX2, and NOS2 are core targets of TWH and key regulators in ROS metabolism. Our molecular docking study demonstrated that bioactive ingredients of TWH such as triptolide, kaempferol and 5,8-Dhmc could bind to these targets with high binding affinity. These findings suggest that the antioxidative effect of TWH may alleviate the symptoms of BD.

Given the limitations of network pharmacology, further biological experiments are required to confirm our findings. Moreover, since all the databases included in our study are based on the previous researches, the discovery of new targets related to TWH for BD may be limited and may not always be timely. Furthermore, the conventional network pharmacology technique is limited to the protein-coding genes, neglecting other gene categories that could also play a key role in BD treatment. At last, the interactions between the bioactive components of TWH were not taken into account in this study and require further assessment.

## 5. Conclusion

We utilized network pharmacology and molecular docking to investigate the effects of TWH bioactive ingredients on target genes related to BD. Through this approach, we identified core targets, including TNF, IFNG, PTGS2, NOS2, VCAM-1, and IL-2, that could potentially play a key role in TWH for treating BD. Furthermore, molecular docking analysis demonstrated that TWH bioactive compounds, such as kaempferol and triptolide, exhibited strong binding affinity towards their respective core targets. Additionally, the antioxidant properties of TWH bioactive compounds were found to contribute to their potential therapeutic benefits in BD. These findings provide a preliminary understanding of the mechanisms behind TWH’s efficacy in managing BD, and offer valuable insights for further research and clinical applications.

## Acknowledgements

We thank Zhen Tian for providing assistance with language editing and manuscript formatting.

## Author contributions

**Conceptualization:** Lihua Ye, Li Wang.

**Data curation:** Changrong Li, Xiaoxia Zhao, WeiHong Ou.

**Formal analysis:** Lihua Ye, Changrong Li.

**Funding acquisition:** Lihua Ye, Mengjie Wan.

**Methodology:** Lihua Ye.

**Supervision:** Mengjie Wan.

**Visualization:** Lihua Ye.

**Writing – original draft:** Lihua Ye, Changrong Li, Xiaoxia Zhao.

**Writing – review & editing:** Lihua Ye, Li Wang, Mengjie Wan.

## Supplementary Material








