# Supplementary material for: Exploring the pharmacological mechanism of Tripterygium wilfordii hook for treatment of Behcet’s disease using network pharmacology and molecular docking
Source: Medicine (Baltimore). 2023 Oct 20;102(42):e34512. doi: 10.1097/MD.0000000000034512 (PMC10589559; doi:10.1097/MD.0000000000034512)
Supplement: Supplementary file 4 [file medi-102-e34512-s004.docx]

Molecular docking results, click the icon below to open.
